# *In-Situ* Cure Monitoring of Wind Turbine Blades by Using Fiber Bragg Grating Sensors and Fresnel Reflection Measurement

**DOI:** 10.3390/s150818229

**Published:** 2015-07-27

**Authors:** Umesh Sampath, Hyunjin Kim, Dae-gil Kim, Young-Chon Kim, Minho Song

**Affiliations:** Division of Electronics and Information Engineering, Chonbuk National University, Jeonju 561-756, Korea; E-mails: sjumesh@jbnu.ac.kr (U.S.); dldpavl@jbnu.ac.kr (H.K.); abcd@jbnu.ac.kr (D.K.); yckim@jbnu.ac.kr (Y.-C.K.)

**Keywords:** cure monitoring, Fresnel reflectivity, fiber Bragg grating, distorted sensors, Gaussian curve fitting

## Abstract

A fiber-optic cure monitoring system is proposed to measure curing status of composite structure such as a large scale wind turbine blade. The monitoring is based on the measurement of Fresnel reflectivity at the optical fiber/epoxy resin interface. The refractive index of epoxy resin varies throughout curing stages, changing the Fresnel reflectivity. The curing status is decided by monitoring the reflected intensity variation. The usage of fiber Bragg grating (FBG) sensor helps to separate the temperature-induced cross effects. A Gaussian curve fitting algorithm was applied to FBG spectra which were distorted in curing procedure. The substantial measurement errors could be minimized by locating the centroids of the Gaussian curve-fitted spectra. From the experiments performed in various isothermal conditions, the proposed system successfully identified the onset of gelation and the completion of curing of epoxy resins.

## 1. Introduction

The usage of composite materials has been examined in various industrial applications. Wide spread adoption of composite materials is because of their unique features such as light weight, high strength, non-corrosiveness, dielectric nature, and chemical resistance compared to natural materials. In recent years, many ongoing researches are trying to improve the materials’ quality for increasing demanding applications such as huge off-shore wind turbine blades which undergo severe fluctuations in loads and environments. The quality and the reliability of thermoset composite materials rely on thermo-kinetic and chemo-rheological properties of polymer, such as degree of cure and viscosity, which are determined by cure cycle parameters [1]. 

Curing refers to the hardening of a polymer material by cross-linking of polymer chains. Despite the wide variety of thermoset resin, their cure behavior is basically identical. The resin viscosity drops initially upon the application of heat that passes through maximum flow. However, it gradually increases as the chemical reactions expand the length and degree of cross-linking. The real-time cure monitoring is essential for the control of manufacturing process of the composite materials. With accurate information about curing status, they can shorten the manufacturing process, and reduce the cost. Furthermore, undesired properties in the final rigid solid, such as exothermal peaks or residual strain gradients, can be alleviated by analyzing and optimizing the process with *in-situ* cure monitoring. For the cure monitoring purposes, the dielectric analysis and the Raman spectroscopy have been widely used [2,3]. Recently, fiber-optic sensors have been drawing greater attention as they provide superior results in cure monitoring. That is because of their inherent properties such as immunity against electromagnetic interference, low cost, and moreover the easiness to be embedded inside the composite materials without any detrimental effects on the host structures [4,5]. We note that most of the previous fiber-optic cure monitoring schemes involve refractive index measurements, interferometric, and fiber Bragg grating (FBG) sensors [6,7,8,9]. Of the techniques, FBGs have advantages over other intensity-based sensors because of multiplexing capability as well as wavelength encoding that enables robust measurement regardless of external environmental perturbations and power fluctuations of light source. The FBG sensors, however, undergo spectral distortions when they are exposed to lateral stresses, which commonly happen in multilayer composite structures. The distortions of Bragg spectra make it difficult to locate the central Bragg wavelength, causing substantial measurement errors which have been reported earlier [10]. Also, temperature sensitive reaction of FBG sensor makes it difficult to decide the curing status in non-isothermal curing conditions.

In this paper, we propose a new sensor model to achieve real time information about polymer resin cure advancement. By combining the Fresnel reflection measurement and the fiber Bragg grating sensors, the more accurate cure status could be obtained even under complicated environments. The measurement accuracy of the spectrally distorted FBG sensor was improved by applying Gaussian curve fitting.

## 2. Principles 

**Figure 1:**
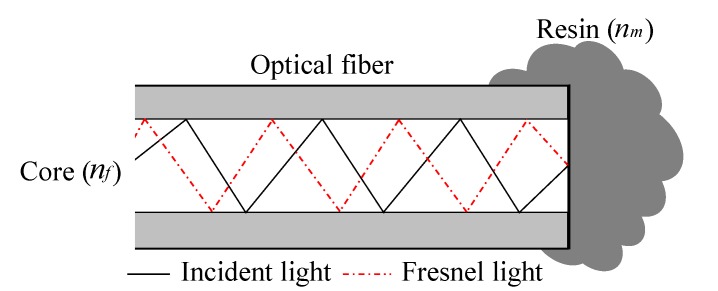
Fresnel reflection at fiber end/resin medium.

In the field of polymers, Lorentz-Lorentz law describes a direct dependence of refractive index and the densities of resin materials by following Equation [11].

(1) $$\frac{n^{2}-1}{n^{2}+2}=\frac{N}{3M\eta }\rho \beta$$

where *n* is the refractive index, η is the free space permittivity, *N* is the Avogadro number, *M* is the molecular weight, ρ and β denotes the density and the polarizability of the resin. Curing is an exothermal polymerization reaction forming an irreversible final product with different physical nature. At initial stage of curing, resin has a low viscosity. With the commencement of polymerization reaction, that is cross-linking between polymer chains, the viscosity increases and attains a gel state. Further cross-linking of polymer chains leads to finally cured product. The whole process is directly related to a continuous change in refractive index and density. Suggested cure monitoring system utilizes the change of Fresnel reflection intensity which is caused by the change of refractive indices of interfacing materials. Figure 1 illustrates the Fresnel reflection at interface of fiber end/resin medium.

The Fresnel reflection coefficients at the fiber end/resin interface for the parallel and the perpendicular polarizations are expressed as following relations

(2) $$r_{p}=\frac{n_{m}\text{cos}\theta_{1}-n_{f}\text{cos}\theta_{2}}{n_{m}\text{cos}\theta_{1}+n_{f}\text{cos}\theta_{2}},\;r_{n}=\frac{n_{f}\text{cos}\theta_{1}-n_{m}\text{cos}\theta_{2}}{n_{f}\text{cos}\theta_{1}+n_{m}\text{cos}\theta_{2}}$$

The refractive indices of the fiber and the resin are denoted as $n_{f}$ and $n_{m}$; $\theta_{1}$ and $\theta_{2}$ are the angles of incidence and reflection, respectively. Assuming the paraxial paths in standard single mode fiber, that is $\theta_{1}\approx \theta_{2}\approx 0$, the Fresnel reflectivity R can be expressed as

(3) $$R={\left|r_{f}\right|}^{2}={\left|r_{m}\right|}^{2}={\left|\frac{n_{f}-n_{m}}{n_{f}+n_{m}}\right|}^{2}$$

The incident laser power, detector gain, coupling coefficient, fiber loss and reflection coefficient compose the Fresnel reflection sensor output.

### 2.1. Fiber Bragg Gratings

In a fiber Bragg grating (FBG), a length of periodic refractive index perturbation reflects a narrow wavelength band called Bragg wavelength

(4) $$\lambda_{B}=2n_{eff}\text{Λ}$$

As in Equation (4), Bragg wavelength depends on effective refractive index of optical fiber core ($n_{eff}$) and spatial period of grating pitch (Λ). When temperature and strain applies to a FBG, the Bragg wavelength shift is given by [12]:

(5) $$\text{Δ}\lambda_{B}=2\left(\text{Λ}\frac{\partial n_{eff}}{\partial \varepsilon }+n_{eff}\frac{\partial \text{Λ}}{\partial \varepsilon }\right)\text{Δ}\varepsilon +2\left(\text{Λ}\frac{\partial n_{eff}}{\partial T}+n_{eff}\frac{\partial \text{Λ}}{\partial T}\right)\text{Δ}T$$

The first and the second term in the right side of Equation (5) indicate the Bragg wavelength shifts by the applied strain and the temperature change, respectively. 

### 2.2. Demodulation of Distorted FBG 

The strain or the temperature information applied to a FBG sensor is encoded to Bragg wavelength shift. Because the measurands are demodulated from the shift, it is important to locate the centroid of Bragg spectrum precisely. One of the most well-known and commercially successful demodulation schemes is tunable band-pass filter demodulation with coverage of large spectral range in distributed sensing [13]. The interrogation concept relies on transforming Bragg wavelength distribution to temporal peak distribution in time domain by using a wavelength-swept band-pass filter and a photo-detection. This technique is simple and cost-effective, because it uses time domain signal processing. However, it suffers from nonlinearity of wavelength scanning against the driving voltage [14]. 

We used a spectrometer demodulation instead of a tunable band-pass filter to overcome the non-linearity issue [15]. A diffraction grating and a photo-diode array (PDA) are the key elements of the spectrometer. Different Bragg wavelengths are diffracted along the different angles as in Equation (6).

(6) $$m\lambda_{B}=d\text{sin}\theta_{m}$$

where m is the diffraction order, $\lambda_{B}$ is the Bragg wavelength, $\theta_{m}$ is the diffraction angle, and d is the pitch of the diffraction grating. With a diffraction grating and a PDA, the information in wavelength domain is transferred to spatial domain, enabling linear measurement of Bragg wavelength regardless of nonlinear wavelength scanning of the tunable filter. 

## 3. Optical Layout

**Figure 2:**
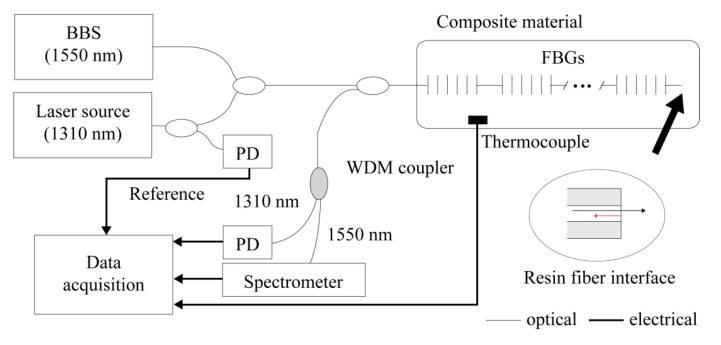
Schematic of cure monitoring system.

Figure 2 shows the experimental setup of the suggested epoxy resin cure monitoring system. The system utilizes two light sources: a broad band source (1550 nm) to lighten the embedded FBGs and a laser source (1310 nm) for Fresnel reflectivity sensing. FBG sensors are connected to the broadband source by two 50:50 single mode couplers, and Bragg wavelengths are demodulated by a spectrometer.

The Bragg spectra reflected from FBGs and the Fresnel reflected light from fiber/resin interface are separated by a wavelength division multiplexing (WDM) coupler, and are collected by a spectrometer and a photo detector (PD), respectively. The intensity-based Fresnel sensor is sensitive to output power fluctuations of light source, changing sensor output regardless of cure status. In order to compensate this error, the Fresnel reflection output is referenced by the tapped output power of the 1310 nm laser source. The referenced sensor output is calculated by

(7) $$P_{cal}=\frac{P_{out}}{P_{ref}}$$

where $P_{out}$, $P_{ref}$ are the sensor output power and the tapped reference power, respectively. 

## 4. Experiments and Results

### 4.1. Sample Preparation 

**Figure 3:**
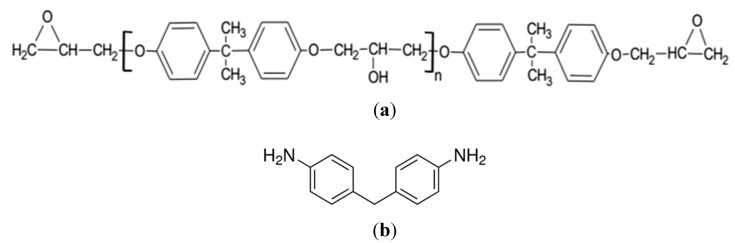
Chemical structures of (**a**) Diglycidyl Ether of Bisphenol-A (DGEBA), and (**b**) 4, 4’ Diamine-Diphenylmetane (DDM).

The polymer base to be monitored is epoxy resin of the type Diglycidyl Ether of Bisphenol-A (DGEBA) which is liquid at room temperature. Curing agent is the 4, 4’ Diamine-Diphenylmetane (DDM) with physical state of white flakes. Figure 3 shows their chemical structures. Epoxy resins are meant for the good adhesive nature, so permanent adhesion between fiber end and resin are expected. 

Table 1 shows the properties of curing agent. The samples for curing epoxy resins were prepared by mixing stoichiometric proportions of 30 parts DDM to 100 parts of resin. Extra care was taken to avoid air bubbles in epoxy which could interrupt clear contact with fiber end face. Glass fiber laminates were used for multilayer composite structure of wind turbine blades.

**Table 1:** Properties of 4, 4’ diamine-diphenylmetane [16].

Curing Agent	DDM
Weight of active hydrogen (g.mol^−1^)	49.5
Peak Melting point ( °C)	94
Molecular weight (g.mol^−1^)	226

### 4.2. Curing at Room Temperature and Isothermal Test

**Figure 4:**
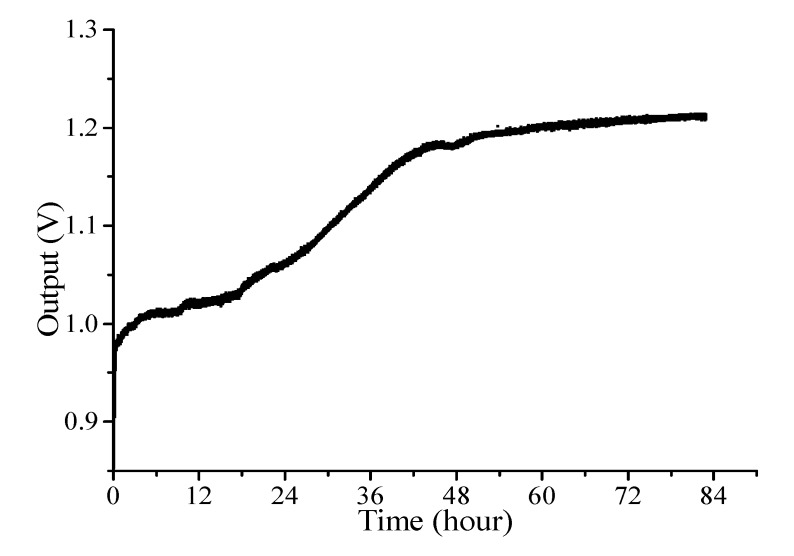
Fresnel reflectivity variation at room temperature.

Curing cycle was carried out at room temperature with the prepared sample. The Fresnel sensor recorded the changes in the Fresnel reflectivity, which is shown in Figure 4 according to time. The sensor output was referenced with the tapped laser power. 

It is clearly seen that, as the cross-linking of polymer proceeds, the amount of Fresnel reflection increased. The Fresnel intensity saturated at about 51 hours after the start of curing procedure. 

**Figure 5:**
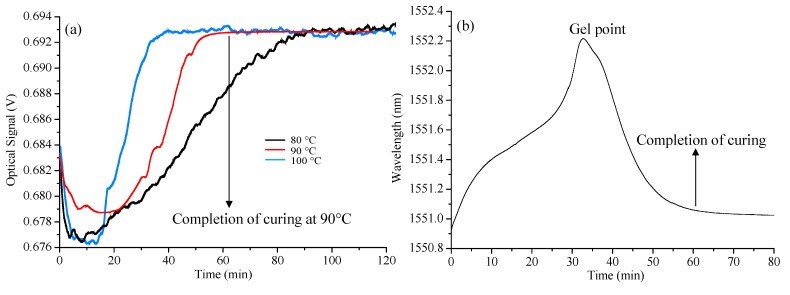
Sensor outputs ((**a**): Fresnel at 80, 90, and 100 °C, (**b**): fiber Bragg grating (FBG) at 90 °C).

Curing tests with the same preparations were carried out in an isothermal chamber with ambient temperatures at 80, 90, and 100 °C. In order to validate the results, the same curing conditions were practiced with the DSC (differential scanning calorimetric) technique performed by Kim *et al.* [17]. Figure 5a shows the results from the isothermal tests. All the sensor outputs show initial decreases as the density of resin mixture reduces with application of external heat. At the initial stage of cross-linking, the outputs increase as the refractive index of resin changes. Finally, fully cured resin holds the refractive index and the density as constant parameters, saturating the Fresnel sensor outputs. Fresnel sensor outputs clearly showed the beginning of cross-linking polymer chains and the completion of curing in real time.

For comparison, the FBG sensor was located in the same sample. Its output was recorded and shown in Figure 5b according to curing time. The ambient temperature was fixed at 90 °C. Being different from the Fresnel outputs, the Bragg wavelength continuously increased till the gelation started. The FBG sensor did not respond to the stress factor in liquid state but was affected only by the temperature change. The advancement of polymer chain cross-linking results in denser networks with higher viscosity and molecular weights in exothermic reaction. When the highly viscous liquid transforms to gel state, the stress caused by epoxy resin shrinkage results in steep decrease of Bragg wavelength, which indicates the onset of gelation. When the epoxy resin is transformed to a rigid solid form, the applied stresses act permanently, fixing the Bragg wavelength. 

**Figure 6:**
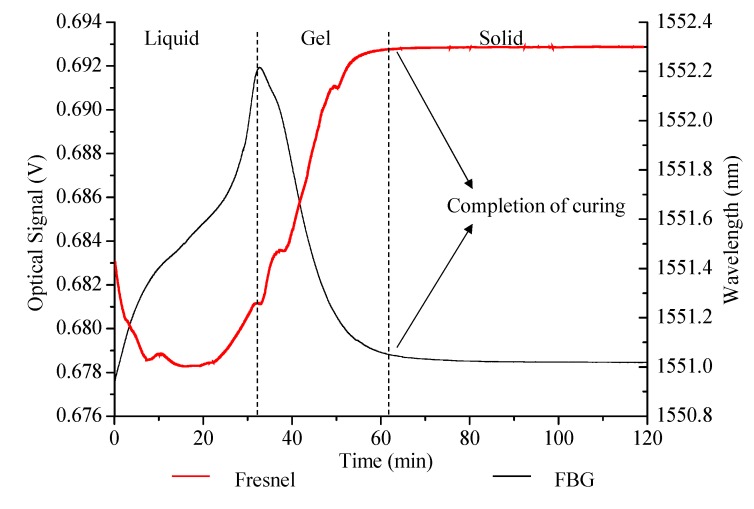
Comparison of Fresnel and FBG signals at 90 °C.

Figure 6, shows the comparison of Fresnel sensor output with the FBG sensor response. The outputs from different sensors match well in time scale indicating the completion of curing at 90 °C in nearly 63 minutes. The indication of gelation is observed by the sudden drop of Bragg wavelength which was induced by the increase in density as cross-linking of polymer chains occurred. Both signals approached to stable outputs, indicating the completion of curing.

**Figure 7:**
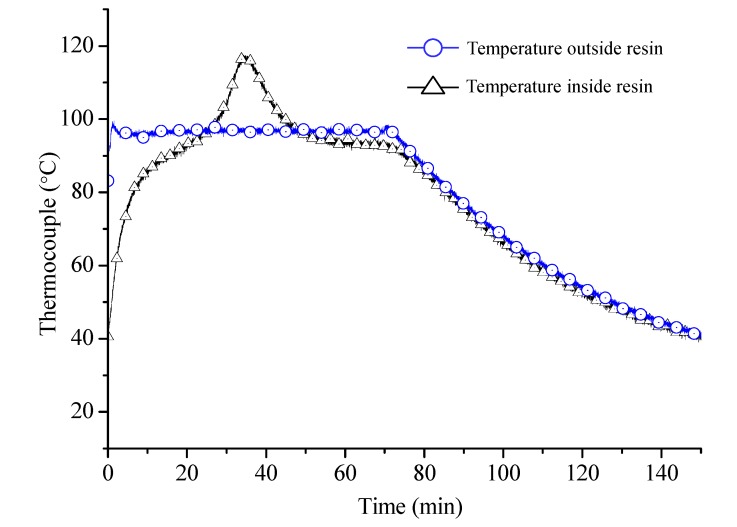
Temperature profiles measured in oven and epoxy resin.

Figure 7, shows the temperature measurement results of isothermal epoxy resin curing process. Two thermocouple sensors were used: one in between the laminates and the other in isothermal chamber. 

Temperature profiles obtained from the thermocouple which was embedded in epoxy resin shows the increase in temperature at first stage to attain the set temperature. In second stage, cross-linking of polymer chains begins with increase in density, increasing the inside temperature of the composite laminate, which is evident from temperature measurements shown in Figure 7. When the cross-linking completes, the temperature decreases to set temperature. This validates that the Bragg wavelength shift in early stage purely depends on temperature factor. 

### 4.3. Gaussian Curve Fitting

**Figure 8:**
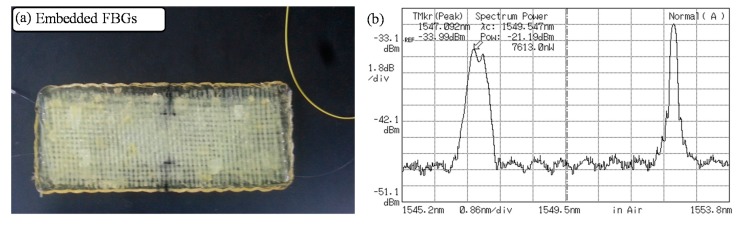
Distortion in Bragg spectrum ((**a**) Cured composite block, (**b**) FBG’s Bragg spectrum (left: distorted FBG)).

Figure 8a shows the sample of cured composite block. In the middle of curing process, the embedded FBG sensors are stressed by the shrinkage of epoxy resin. Because of the irregular strain fields, the reflection behaviors of FBG sensors change, distorting the Bragg reflection spectra as in Figure 8b. The central peak is difficult to be located in the distorted spectrum, leading to substantial measurement errors.

**Figure 9:**
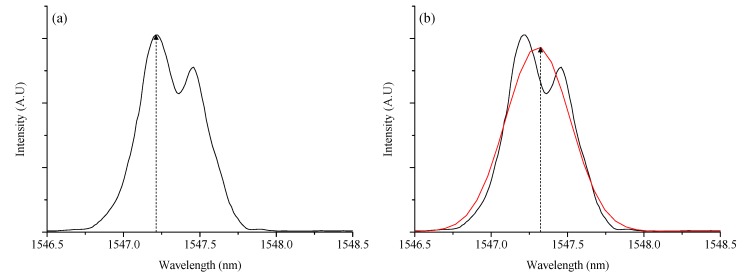
Center positioning of FBG sensor spectrum ((**a**): Without Gaussian curve fitting, (**b**): With Gaussian curve fitting).

In order to minimize the errors, we applied a Gaussian curve fitting to the distorted spectrum [10]. Figure 9 shows the example of Gaussian curve fitting. It is a spectrometer readout of an FBG spectrum and its Gaussian-fitted curve. There is ~0.108 nm of difference in the central peak positions, which corresponds to temperature measurement error of 10 degrees. In large scale composite structures, for example a wind turbine blade, permanent distortions in FBG spectra should happen. It will be useful to apply Gaussian curve fitting not only in manufacturing process but also in-field condition monitoring uses to reduce measurement errors. 

## 5. Conclusions

A fiber-optic cure monitoring system is proposed to measure the curing status of composite structures. It is evident from the studies performed and reported in this paper that the Fresnel and FBG sensors can be used efficiently as a real-time cure monitoring sensors. The inception of gelation and the completion of curing could be successfully detected by combining both the outputs without ambiguity caused by temperature effect. With experimental results performed in various isothermal conditions, it was shown that an optimal curing condition could be found. The FBG sensors used in cure monitoring can be used for in-field condition monitoring of the host structure by applying Gaussian curve fitting to the distorted Bragg spectra. This fiber-optic cure monitoring scheme would be useful in deciding upon optimal curing conditions and, later, condition monitoring uses.

## References

[1] Kenny J.M., Apicella A., Nicolais L. (1989). A model for the thermal and chemoreological behavior of thermosets: 1. Processing of epoxy based composites. Polym. Eng. Sci..

[2] Mijovic J., Kenny J., Maffezzoli A., Trivisano A., Bellucci F., Nicolais L. (1993). The principles of dielectric measurements for *in situ* monitoring of composite processing. Compos. Sci. Technol..

[3] Lyon R.E., Chike K.E., Angel S.M. (1994). *In situ* cure monitoring of epoxy resins using fiber-optic Raman spectroscopy. J. Appl. Polym. Sci..

[4] Afromowitz M.A. (1988). Fiber optic polymer cure sensor. Lightwave Technol. J..

[5] Lam K.Y., Afromowitz M.A. (1995). Fiber-optic epoxy composite cure sensor. I. Dependence of refractive index of an autocatalytic reaction epoxy system at 850 nm on temperature and extent of cure. Appl. Opt..

[6] Cusano A., Breglio G., Giordano M., Calabro A., Cutolo A., Nicolais L. (2000). An optoelectronic sensor for cure monitoring in thermoset-based composites. Sens. Actuators. A: Phys..

[7] Cusano A., Cutolo A., Giordano M., Nicolais L. (2003). Optoelectronic refractive index measurements: Application to smart processing. IEEE Sens. J..

[8] Leng J.S., Asundi A. (2002). Real-time cure monitoring of smart composite materials using extrinsic Fabry-Perot interferometer and fiber Bragg grating sensors. Smart Mater. Struct..

[9] Murukeshan V.M., Chan P.Y., Ong L.S., Seah L.K. (2000). Cure monitoring of smart composites using Fiber Bragg Grating based embedded sensors. Sens. Actutors A: Phys..

[10] Lee H.W., Park H.J., Lee J.H., Song M. (2007). Accuracy improvement in peak positioning of spectrally distorted fiber Bragg grating sensors by Gaussian curve fitting. Appl. Opt..

[11] Antonucci V., Giordano M., Cusano A., Nasser J., Nicolais L. (2006). Real time monitoring of cure and gelification of a thermoset matrix. Compos. Sci. Technol..

[12] Othonos A., Kalli K. (1999). Properties of Fiber Bragg Gratings. Fiber Bragg Gratings: Fundamentals and Applications in Telecommunications and Sensing.

[13] Park H.J., Song M. (2008). Linear FBG temperature sensor interrogation with Fabry-perot ITU multi-wavelength reference. Sensors.

[14] Jiang B., Zhao J., Qin C., Huang Z., Fan F. (2011). An optimized strain demodulation method based on dynamic double matched fiber Bragg grating filtering. Opt. Lasers Eng..

[15] Kim H., Song M. (2013). A fiber laser spectrometer demodulation of fiber Bragg grating sensors for measurement linearity enhancement. J. Opt. Soc. Korea.

[16] Costa M.L., Pardini L.C., Rezende M.C. (2005). Influence of aromatic amine hardeners in the cure kinetics of an epoxy resin used in advanced composites. Mater. Res..

[17] Kim H., Char K. (1999). Dielectric changes during the curing of epoxy resin based on the Diglycidyl Ether of Bisphenol A (DGEBA) with diamine. Bull. Korean Chem. Soc..

